# Prognosis and risk factors for cardiac valve calcification in Chinese end-stage kidney disease patients on combination therapy with hemodialysis and hemodiafiltration

**DOI:** 10.1080/0886022X.2022.2032742

**Published:** 2022-02-15

**Authors:** Jian-qiong Xiong, Xue-mei Chen, Chun-ting Liang, Wen Guo, Bai-li Wu, Xiao-gang Du

**Affiliations:** a Department of Nephrology, The First Affiliated Hospital of Chongqing Medical University, Chongqing, China; b Hospital, Chongqing University, Chongqing, China; c Emergency Department, The First Affiliated Hospital of Chongqing Medical University, Chongqing, China; d Department of Nephrology, Longchang People’s Hospital, Neijiang, Sichuan, China

**Keywords:** End-stage kidney disease, hemodialysis, hemodiafiltration, cardiac valve calcification, vascular calcification

## Abstract

**Background:**

Cardiac valve calcification (CVC) is an important risk factor for cardiovascular complications. However, limited data are available concerning the prevalence, clinical features and risk factors for CVC in end-stage kidney disease (ESKD) patients. In this study, we aimed to assess these parameters in Chinese ESKD patients receiving combination therapy with hemodialysis and hemodiafiltration.

**Methods:**

We conducted a cross-sectional study on 293 ESKD patients undergoing combination therapy of hemodialysis and hemodiafiltration at the First Affiliated Hospital of Chongqing Medical University from October 2014 to December 2015. CVC was evaluated *via* echocardiography.

**Results:**

ESKD patients with CVC had a higher prevalence of diabetes mellitus, aortic and/or coronary artery calcification, arrhythmia, heart failure and coronary heart disease; increased systolic, diastolic and pulse pressure; longer duration of hemodialysis and hypertension; reduced hemoglobin, albumin and high-density lipoprotein cholesterol levels; and increased serum calcium and calcium-phosphorus product levels compared with those without CVC. Logistic regression analysis showed that increased dialysis duration (*p* = 0.006, OR = 2.25), serum calcium levels (*p* = 0.046, OR = 2.04) and pulse pressure (*p* < 0.001, OR = 3.22), the presence of diabetes (*p* = 0.037, OR = 1.81) and decreased serum albumin levels (*p* = 0.047, OR = 0.54) were risk factors for CVC. The correlation analysis indicated a significantly increased CVCs prevalence with an increase prevalence of heart failure, aortic and coronary artery calcification.

**Conclusions:**

CVC represents a common complication and a danger signal for cardiovascular events in ESKD patients undergoing combination therapy of hemodialysis and hemodiafiltration. The presence of diabetes, increased pulse pressure, long dialysis duration, hypoalbuminemia and high serum calcium levels were independent risk factors for CVC.

## Introduction

Individuals with end-stage kidney disease (ESKD) are at increased risk for cardiovascular disease (CVD), which has been considered an important predictor for all-cause mortality and cardiovascular mortality [1,2]. Cardiac valve calcification (CVC) is tightly associated with cardiovascular complications [3]. Epidemiological and clinical studies have demonstrated that calcification in the vasculature or tissue is common in ESKD patients, and greater than half of the adult hemodialysis patients have evidence of CVC [4,5].

The pathophysiology of CVC is multifactorial and not completely understood. Age, sex, smoking, and primary diseases, such as hypertension or diabetes mellitus, are traditional risk factors for CVC [6]. Inflammation and malnutrition, including anemia and hypoproteinemia, which have been shown to be associated with a high risk of cardiovascular complications in chronic kidney disease (CKD) [7], may play important roles in CVC. The kidney is important for maintaining the balance between calcium and phosphorus metabolism. CKD is typically accompanied by calcium and phosphorus metabolism disorders, which increase the risk of vascular or tissue calcification [8]. However, in Chinese ESKD patients undergoing renal replacement therapy (RRT), CVC remains uncharacterized, and the underlying mechanisms and risk factors remain undefined. The aim of the study was to investigate the incidence, risk factors and clinical features of CVC in Chinese ESKD patients receiving RRT to prevent CVC and provide evidence for therapeutic regimen adjustment.

## Materials and methods

### Study subjects

**Figure 1.**
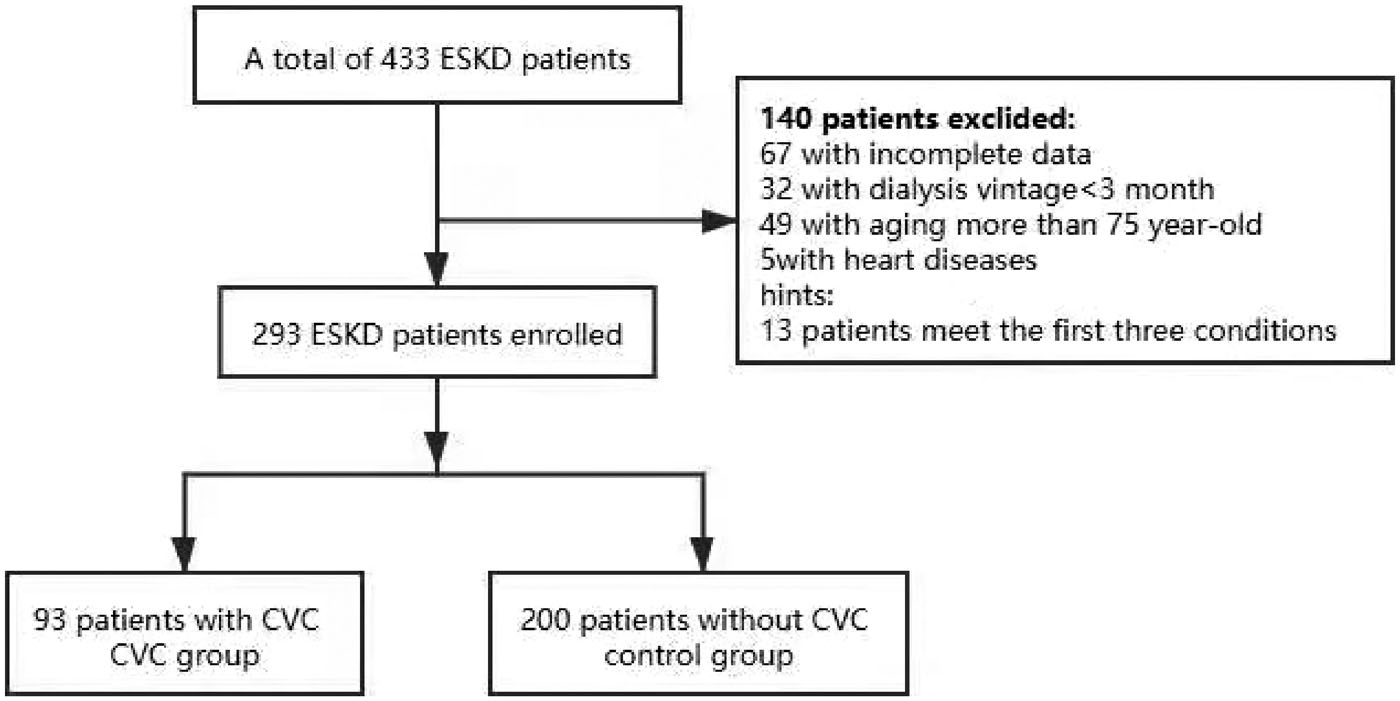
Flow chart of study design.

**Figure 2.**
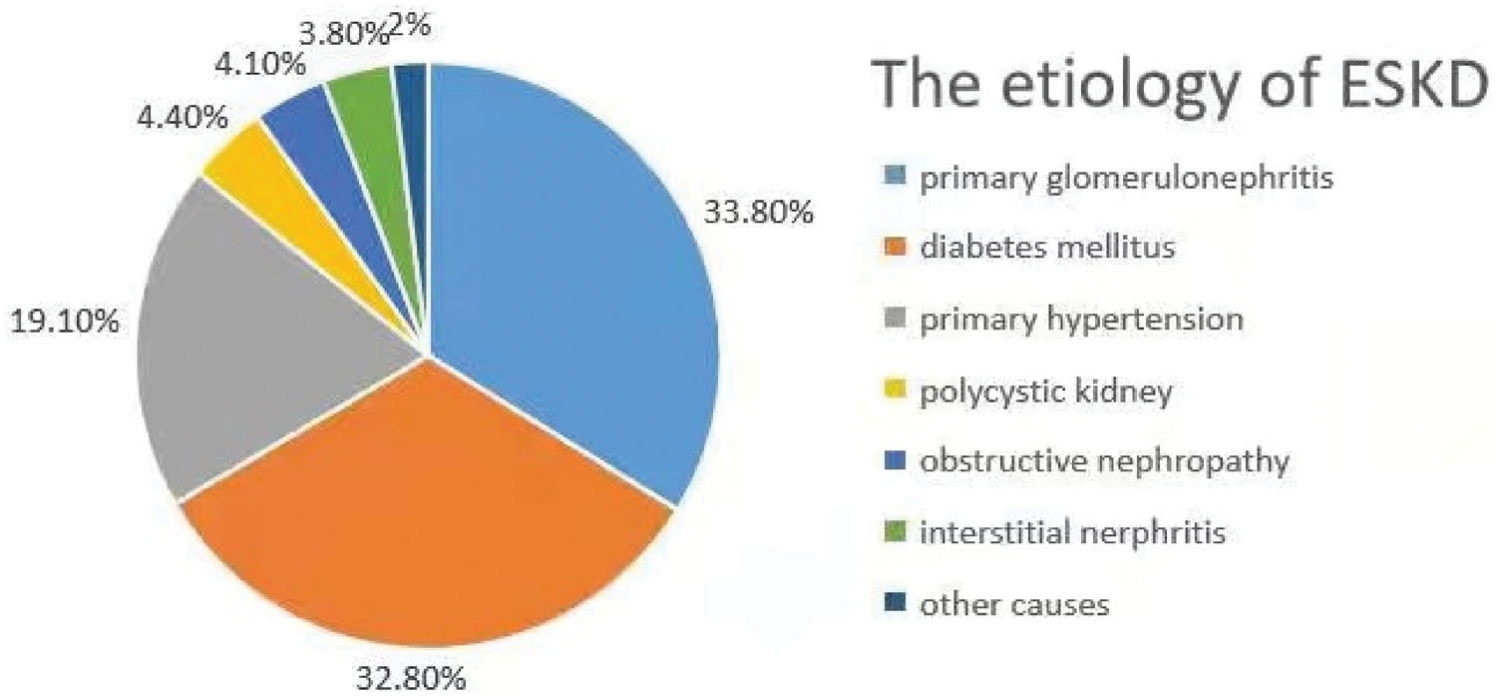
Etiology of end-stage kidney disease patients.

We conducted a cross-sectional study of ESKD patients who had received more than 3 months of combination therapy with hemodialysis and hemodiafiltration in the dialysis center of the First Affiliated Hospital of Chongqing Medical University from October 2014 to December 2015. The exclusion criteria were as follows: dialysis duration less than 3 months, older than 75 years of age, congenital heart disease, infective endocarditis, rheumatic heart disease and incomplete data. In total, 293 patients were enrolled in this study, including 160 males and 133 females aged 43–74 years with an average age of 64 ± 7.2 years. Nighty-three ESKD patients with CVC confirmed by echocardiography were assigned to the CVC group. Two hundred ESKD patients without CVC were assigned to the control group (Figure 1). The etiologies of 293 ESKD patients are presented in a pie chart (Figure 2).

### Treatment

In total, 293 patients underwent regular hemodialysis for 4 h twice a week and online hemodiafiltration for 4 h once a week using a Helixone Haemodlafilter FX 800 (Surface Are: 1.8 m^2^) or NIPRO Hollow fiber dialyzer FB-150U (Surface Area: 1.5 m^2^) combined with intermittent hemoperfusion once every month using the Jafron HA130 disposable hemoperfusion system. Blood purification was conducted on a Fresenius Medical Care 4008s or GAMBRO AK96 hemodialysis machine. Autogenous arteriovenous fistulas were the preferred route for vascular access followed by arteriovenous grafts or venous catheters. The patient’s blood was extracted at a blood flow rate of 230–280 mL/min. All dialysate calcium concentrations were not greater than 1.25–1.50 mmol/L. All patients received conventional drug therapy, such as antihypertensive treatment with calcium channel blockers (CCBs), angiotensin-converting enzyme inhibitors (ACEIs), angiotensin receptor blockers (ARBs), calcium and phosphorus-regulating treatment with a calcium-containing phosphorous binder or alfacalcidol tablets, anti-anemia treatment with iron preparations or recombinant human erythropoietin (EPO).

### Clinical data collection

General information, including age, sex, cigarette smoking, duration of hemodialysis and hypertension, diabetes and clinical manifestations, including cardiovascular complications and cerebrovascular diseases (CVDs) was collected. Systolic, diastolic and pulse pressures were measured before hemodialysis at 2-week intervals and were averaged over 3 months.

Laboratory variables, including blood hemoglobin, serum albumin, calcium, phosphorus, intact parathyroid hormone (iPTH), lipids and high sensitivity C-reactive protein (hs-CRP), were collected before hemodialysis at the time of enrollment. Calcium was corrected for serum albumin levels <40 g/L as follows [9]:

Corrected calcium (mmol/l)=calcium (mmol/l)+0.2×[4−serum albumin (g/dl)]

### Echocardiography

All subjects underwent echocardiography on a Philips IE33 echocardiograph equipped with an S5–1 PureWave array probe (frequency 1.7/3.3 MHz) to verify the presence of CVC. CVC was defined as a strong echo signal of greater than 1 mm on one or more cusps of the aortic valve, mitral valve or mitral annulus [10].

### Computer tomography scan

To determine vascular calcification, 282 ESKD patients (90 patients in the CVC group and 192 patients in the nCVC group) underwent chest and abdominal computed tomography (CT) scans on a SOMATOM Perspective scanner, and a few patients with suspected acute coronary syndrome underwent coronary CT angiography. A calcified plaque in the coronary artery and aorta was considered present if the CT value was greater than 130 [11].

### Statistical analysis

Statistical analysis was performed using SPSS 21.0 statistical software. Data were assessed a normal distribution using the Kolmogorov-Smirnoff test. All numeric data with a normal distribution were presented as the means ± SD and analyzed using the Student's *t*-test. Numeric variables with skew distribution were expressed as median (interquartile range) and compared using the Mann-Whitney *U* test. Categorical variables were expressed as percentages and analyzed using the chi-square test. The risk factors associated with CVC were identified by binary logistic regression analysis. The relation between CVC and its associated complications was evaluated using Spearman’s correlation analysis. *p* < 0.05 was considered statistically significant.

## Results

### Prevalence of cardiac valve calcification in ESKD patients

In our study, CVC was found in 93 of 293 patients (31.7%), aortic valve calcifications (AVC) in 68, mitral valve calcifications (MVC) in 37, and both valve calcifications (MVC + AVC) in 12 patients.

### Basic clinical characteristics of ESKD patients with CVC

Of 293 ESKD patients enrolled in this study, the 93 patients with CVC (CVC group) included 55 males and 38 females ranging in age from 44 to 74 years (64.0 ± 7.0 years old). Two hundred ESKD patients without CVC (nCVC group) included 105 males and 95 females ranging in age from 43 to 74 years (64.0 ± 7.0 years old).

We explored the risk factors for CVC in ESKD patients and found significantly increased diabetes mellitus prevalence, systolic pressure, diastolic pressure and pulse pressure, dialysis vintage and hypertension duration in the CVC group compared with the nCVC group (*p* < 0.05). No differences in age, sex, history of smoking or hypertension prevalence were noted between the two groups (Table 1).

**Table 1.** The basic clinical characteristics of ESKD patients with CVC and without CVC.

	CVC (*n* = 93)	nCVC (*n* = 200)	* P*
Age, years	64.0 ± 7.0	64.0 ± 7.0	0.869
Male, *n* (%)	55 (59.1%)	105 (52.5%)	0.288
Smoking, *n* (%)	37 (39.8%)	78 (39.0%)	0.898
Diabetes mellitus, *n* (%)	53 (57.0%)	89 (44.5%)	0.046
Hypertension duration, years	10 (6, 13)	8 (3, 11)	0.027
Hypertension, *n* (%)	92 (98.9%)	195 (97.5%)	0.833
SP, mmHg	160 ± 22	142 ± 20	<0.001
DP, mmHg	83 ± 14	73 ± 11	<0.001
PP, mmHg	77 ± 14	69 ± 17	<0.001
Dialysis duration, months	32 (8, 72)	19 (7, 43)	0.038

SP: systolic pressure; DP: diastolic pressure; PP: pulse pressure.

### Nutritional and inflammatory status in ESKD patients with CVC

Nutritional and inflammatory status is an important influential factor for CVC occurrence; therefore, we explored the correlation of nutritional and inflammatory parameters with CVC in ESKD patients. As shown in Table 2, reduced blood hemoglobin levels and serum albumin and high-density lipoprotein cholesterol (HDL-C) levels were noted in the CVC group compared with the nCVC group (*p* < 0.05). No significant difference in serum total cholesterol (TC), high-density lipoprotein cholesterol (LDL-C), triglyceride (TG), and high-sensitivity C-reactive protein (hs-CRP) levels were noted between the two groups.

**Table 2.** Nutritional and inflammatory status in ESKD patients with and without CVC.

	CVC (*n* = 93)	nCVC (*n* = 200)	*P*
Hemoglobin, g/L	95.6 ± 25.3	104.4 ± 22.7	0.005
Albumin, g/L	36.4 ± 5.5	37.9 ± 5.2	0.027
TC, mmol/L	3.63 (2.97, 4.26)	3.76 (3.00, 4.47)	0.318
LDL-C, mmol/L	2.25 ± 0.79	2.17 ± 0.89	0.407
HDL-C, mmol/L	1.01 (0.82, 1.21)	1.06 (0.87, 1.35)	0.044
TG, mmol/L	1.16 (0.90, 1.57)	1.51 (0.89, 2.03)	0.442
hs-CRP, mg/L	10.89 (3.41, 20.00)	10.28 (2.15, 20.00)	0.365

TC: total cholesterol; LDL-C: low density lipoprotein cholesterol; HDL: high-density lipoprotein cholesterol; TG: triglycerides; hs-CRP: high sensitivity C-reactive protein.

### The calcium-phosphorus metabolism state in ESKD patients with CVC

Alterations in calcium-phosphorus metabolism are associated with an increased risk of CVC. In this study, we analyzed the changes in calcium-phosphorus metabolism-associated parameters in ESKD patients and found that the levels of serum calcium, phosphorus, calcium-phosphorus product and iPTH were 2.26 ± 0.25 mmol/L, 1.72 ± 0.65 mmol/L, 3.65 (2.79, 4.69) mmol/L, 268.80 (129.85, 316.45) pg/ml, respectively. The levels of serum calcium and calcium-phosphorus product in the CVC group were greater than those in the nCVC group (*p* < 0.05). No significant differences in serum phosphorus and iPTH levels were observed between the two groups (Table 3).

**Table 3.** Calcium-phosphorus metabolism state in ESKD patients with and without CVC.

	CVC (*n* = 93)	nCVC (*n* = 200)	*P*
Calcium, mmol/L	2.32 ± 0.27	2.23 ± 0.24	0.005
Phosphorus, mmol/L	1.76 ± 0.55	1.71 ± 0.70	0.461
Calcium × phosphorus product, mmol/L	4.03 (2.92, 4.92)	3.40 (2.67, 4.64)	0.029
iPTH, pg/ml	268.8 (109.9, 307.2)	271.4 (134.7, 337.5)	0.277

iPTH: intact parathyroid hormone.

### Risk factors for CVC in ESKD patients analyzed by binary logistic regression

Predictor variables with *p* < 0.10 in univariate analysis were included in a binary logistic regression model with backward selection. According to the mean values of our patients with CVC, we chose 36 months as cutoff values for the dialysis duration. In the laboratory of our hospital, the lower normal limit of serum calcium levels is 2.11 mmol/L, we used 2.11 mmol/L as a cutoff point for serum calcium value. As the mean values (±SD) of pulse pressure for all our ESKD patients was 72 (±16) mmHg, we chose 72 mmHg as cutoff values for pulse pressure. It is reported serum albumin concentration below 40 g/L was associated with poor outcomes in hemodialysis patients [12], we used 40 g/L as the cutoff serum albumin value. As shown in Table 4, long dialysis vintage (*p* = 0.006, OR = 2.25), the presence of diabetes (*p* = 0.037, OR = 1.81), low serum albumin levels (*p* = 0.047, OR = 0.54), high serum calcium levels (*p* = 0.046, OR = 2.04) and pulse pressure (*p* < 0.001, OR = 3.22) were independent risk factors for CVC in ESKD patients.

**Table 4.** Independent risk factors for CVC in ESKD patients.

	OR (95% CI)	*P*
Dialysis duration ≥36 months (versus <36 months)	2.25 (1.26, 4.02)	0.006
Calcium ≥2.11mmol/L (versus <2.11mmol/L)	2.04 (1.01, 4.12)	0.046
Presence of diabetes mellitus (versus absence)	1.81 (1.04, 3.15)	0.037
Pulse pressure >72mmHg (versus ≤72mmHg)	3.22 (1.85, 5.59)	<0.001
Albumin ≥40g/L (versus <40g/L)	0.54 (0.29, 0.99)	0.047

### Vascular calcification in ESKD patients with CVC

**Figure 3.**
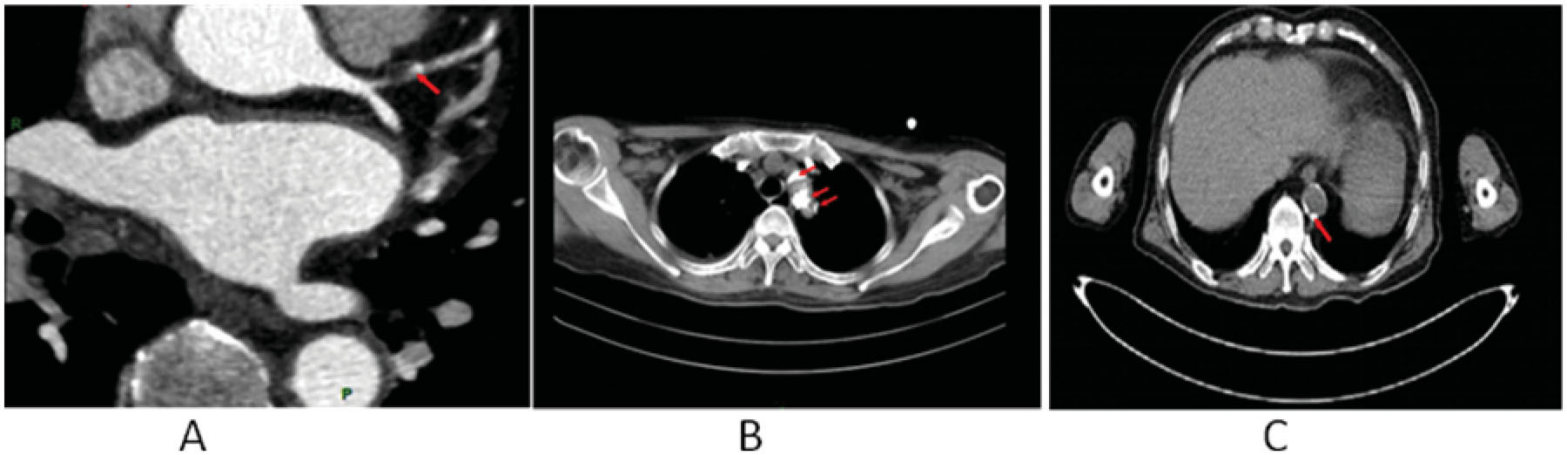
Calcification in the coronary artery and aorta in ESKD patients. Representative calcification in the left anterior descending branch of coronary artery (red arrow) (A), thoracic aorta (red arrow) (B) and abdominal aorta (red arrow) (C).

Vascular calcification is a frequent complication of ESKD. In total, 76.2% of ESKD patients (*n* = 215) had different degrees of calcification in the aortic and/or coronary arteries (Figure 3). The incidence of aortic artery calcification, coronary artery calcification, and both aortic and coronary artery calcification in CVC patients was greater than that in nCVC patients (*p* < 0.001) (Table 5).

**Table 5.** Vascular calcification in ESKD patients with and without CVC.

	CVC (*n* = 90)	nCVC (*n* = 192)	*P*
Aortic calcification, *n* (%)	77 (85.6%)	114 (59.3%)	<0.001
Coronary artery calcification, *n* (%)	59 (65.1%)	72 (37.4%)	<0.001
Both aortic and coronary artery calcification, *n* (%)	50 (55.6%)	57 (29.7%)	<0.001

### Cardio-cerebrovascular complications in ESKD patients with CVC

Information on cardio-cerebrovascular events was collected in ESKD patients. The results showed that the incidence of arrhythmia, heart failure, and coronary heart disease was higher in the CVC group compared with the nCVC group (*p* < 0.05). However, no difference in cerebrovascular complications was found between the two groups (Table 6).

**Table 6.** Cardio-cerebrovascular complications in ESKD patients with and without CVC.

	CVC (*n* = 93)	nCVC (*n* = 200)	*P*
Arrhythmia, *n* (%)	24 (25.8%)	29 (14.5%)	0.019
Heart failure, *n* (%)	16 (17.2%)	14 (7.0%)	0.003
CHD, *n* (%)	43 (46.2%)	65 (32.5%)	0.023
CVD, *n* (%)	19 (20.4%)	37 (18.5%)	0.696

CHD: coronary heart disease; CVD: cerebrovascular disease.

We further used Spearman’s correlation analysis to determine the relationship between CVC and cardiovascular complications in ESKD patients. Our results showed a significantly increased CVC prevalence with an increase prevalence of aortic calcification, coronary artery calcification and heart failure in ESKD patients (*p* < 0.05). However, a relationship between CVC and arrhythmia or CHD was not found (Table 7).

**Table 7.** The relationship between CVC and cardiovascular complications.

	*r*	*P*
Aortic calcification	0.261	<0.001
Aortic and coronary calcification	0.247	<0.001
Coronary calcification	0.248	<0.001
Arrhythmia	0.071	0.229
Heart failure	0.157	0.007
CHD	0.019	0.748

CHD: coronary heart disease.

## Discussion

### CVC is a common complication in ESKD patients

Vascular or tissue calcification is a highly prevalent condition at all stages of CKD. It has been reported that two-thirds of adult hemodialysis patients have electron beam computed tomographic evidence of coronary artery calcification (CAC) and that greater than half exhibit cardiac valve calcification [4,13]. Our results indicate that calcification of vascular and heart valves is a frequent finding among ESKD patients undergoing combination therapy of hemodialysis and hemodiafiltration. A total of 76.2% of our RRT patients had vascular calcification, including aortic calcification (67.7%) and coronary artery calcification (46.5%), and 31.7% had calcification of either mitral or the aortic valve or both. Aortic valve calcification exhibited an increased prevalence compared with mitral valve calcification. The prevalence of mitral VC (39.8%) in our RRT patients was similar to that of hemodialysis (HD) patients reported in the literature, ranging from 10 to 40% [14–17]. However, the prevalence of aortic VC (73.1%) was much higher than that observed in HD patients, ranging from 28 to 55% [4,18]. A high ratio of hypertension (98.9%) and a long duration of hypertension (median duration of hypertension = 10 years) may increase the cardiac afterload and subsequently increase mechanical stress on the valve cusps, hence increasing the risk of aortic VC. Further comparative studies are needed to determine whether hypertension is indeed associated with increased incidence of aortic VC.

### Risk factors for CVC in ESKD patients

Hypertension is one of the traditional risk factors for CVC [19]. Our present study showed a long history of hypertension and higher systolic pressure, diastolic pressure and pulse pressure in ESKD patients with CVC compared with those without CVC, and pulse pressure was identified as an independent risk factor for CVC based on regression analysis.

It is observed vascular calcification, an important component of the atherosclerosis process is prevalent in ESKD patients [20,21]. The deficiency of calcification inhibitors may be the mechanism of vascular calcification. Wang *et al.* found lower matrix-Gla protein (MGP, the first calcification inhibitor) levels were associated with increased risk for coronary artery calcification and cardiovascular events in patients with CKD stage 3–5 [22]. However, Sevinc’s study showed that in stage 2–5 CKD patients who did not require dialysis treatment, there was no correlation between MGP and vascular calcification as expressed with carotid intima-media thickness, whereas the level of Fetuin-A (a major inhibitor of calcium and phosphate precipitation) began to decline from the early stages of CKD, and Fetuin-A levels were negatively correlated to vascular calcification [23]. Large-scale studies on vascular calcification inhibitors are further needed. In this study, we also found that ESKD patients with CVC had a higher incidence of vascular calcification (95.6%) than those without CVC (67.2%), and the prevalence of aortic or coronary artery calcification in the CVC group was 1.44–1.74 times higher than that in the nCVC group. Spearman’s correlation analysis showed that aortic calcification and coronary artery calcification were positively correlated with CVC. Vascular calcifications often develop in two distinct layers of arteries: intimal and medial. Evidence suggests that ESKD patients are major disproportionately affected by medial calcification despite limited intimal calcification [24]. Arterial medial calcifications, which are characterized by vascular stiffening and arteriosclerosis, are related to augmented vascular resistance, decreased vascular compliance, and increased systemic blood pressure and pulse pressure. Furthermore, arterial medial calcifications often occur in diabetic patients as a component of diabetic macroangiopathy. Evidence indicates that arterial medial calcification in diabetes is an active, cell-mediated process that is similar to that observed in ESKD patients [25,26]. In addition, vascular calcification is more serious in ESKD patients with diabetes, and fasting hyperglycemia could be used as a predictor of vascular calcification in ESKD patients [24]. In the present study, we found an increased prevalence of diabetes in ESKD patients with CVC, and regression analysis also showed that the presence of diabetes was an independent risk factor for CVC.

A study [27] showed that the stability of hemodynamics was important for maintaining normal cardiac valve function. Blood pressure is associated with extracellular hydration status in HD patients. Between HD treatments, most HD patients will accumulate volume due to their daily food and fluid intake. Then, the majority of this volume will be removed by hemodialysis in a short period of time (typically 4 h per session, thrice-weekly). Rapid changes in volume status could cause blood pressure fluctuations, which may lead to endothelial cell and stromal cell lesions, inflammation, eventually resulting in CVC. Our study revealed that long dialysis duration was a risk factor for CVC. In addition, Arjona Barrionuevo *et al.* also found CVC was positively correlated with dialysis vintage in ESKD patients [20].

In ESKD patients, inflammation and malnutrition are often considered together. Our data showed that ESKD patients with CVC had lower levels of hemoglobin and albumin, and hypoalbuminemia was an independent risk factor for CVC, suggesting that malnutrition might play a role in the development of CVC in ESKD patients. Similar findings were reported in Plytzanopoulou’s study [7].

Lipid metabolic disorders represent important risk factors for cardiovascular and cerebrovascular disease. Our data showed no significant difference in serum TC, LDL-C and TG levels between the CVC group and the nCVC group. In fact, serum lipid levels do not completely reflect the deposition of lipids in tissue [28]. Furthermore, ESKD patients commonly present reduced appetite and increased catabolism, leading to a malnutrition state, which can reduce serum lipid levels. It is reported lower serum cholesterol level was a risk factor for all-cause death in Western hemodialysis patients, lipid-adjusting drugs could not delay the process of vascular calcification effectively in ESKD patients, and reduce cardiovascular complications and mortality [28–30]. By contrast, in the Japanese hemodialysis population, high non-high-density lipoprotein cholesterol level was a risk factor for cardiovascular death [31,32]. Liu *et al.* found systemic inflammation and malnutrition status could lower the effect of cholesterol resulting in the inverse association of total cholesterol level with cardiovascular complications and death in dialysis patients [33]. Consequently, additional research is required to identify the association of lipids with CVCs in ESKD patients in the absence of inflammation/malnutrition. HDL can transport cholesterol from peripheral tissue and cells to the liver, reduce the deposition of cholesterol in vascular tissues and prevent the development of atherosclerosis [29,33]. Our data revealed much lower peripheral serum HDL levels in ESKD patients with CVC, indicating that HDL might have a protective effect against CVC. Although a state of chronic inflammation often exists in ESKD patients receiving MHD due to increased production of proinflammatory cytokines, chronic and recurrent infections, intestinal dysbiosis, acidosis and biocompatibility of the dialysis membrane [34], the present study did not indicate a significant difference in hs-CRP levels between the CVC group and the nCVC group. These results indicate that inflammation may not be an independent factor for CVC in ESKD patients.

ESKD can lead to hypocalcemia and/or hyperphosphatemia, stimulating excessive PTH secretion, causing secondary hyperparathyroidism, further exacerbating calcium and phosphate imbalance, forming a vicious circle, causing metabolic bone disease and calcification of arteries and other tissues. In this study, we did not find hypocalcemia or high levels of PTH in ESKD patients due to treatment with calcium and vitamin D analogs. Although these patients were treated with phosphorus binders, serum phosphorus levels remained greater than the normal limit. Long-term exposure to a high calcium-phosphorus environment could prompt the transformation of vascular smooth muscle cells into an osteochondrogenic phenotype, reducing the differentiation of monocytes and macrophages into osteoclast-like cells in the vessel wall and the imbalance between mineral deposition and resorption in the vasculature and thereby contributing to vascular calcification in ESKD patients [35,36]. We also found that the levels of serum calcium and calcium-phosphorus products in CVC patients were significantly elevated compared with those in the nCVC group, and further logistic regression analysis showed that a high level of serum calcium was an independent risk factor for CVC. However, the present study did not indicate differences in serum phosphorus and iPTH levels between the ESKD with CVC group and the ESKD without CVC group. The use of calcium-containing phosphorous binders and alfacalcidol tablets can influence the serum calcium, phosphorus and iPTH levels and vascular calcification. Further studies are required to assess the role of phosphorus in CVC progression in ESKD patients.

### The predicted value of CVC in cardio-cerebrovascular complications in ESKD patients

Cardio-cerebrovascular events are the most important cause of death in ESKD patients. A meta-analysis showed that the presence of calcification in any arterial wall is associated with an increased risk for cardio-cerebrovascular events and cardiovascular mortality (2.21-fold increased risk for stroke, 3.41-fold for cardiovascular events and 3.94-fold for cardiovascular mortality) [37]. This study showed that ESKD patients with CVC exhibited an increased prevalence of artery calcification and a positive correlation between CVC and artery calcification. CVC is associated with valve dysfunction, myocardial ischemia, conduction defects and infective endocarditis, which can contribute to arrhythmia, heart failure, and even to sudden death [38]. This study showed that ESKD patients with CVC had 1.4–2.5 times increased prevalence of arrhythmia, heart failure and CHD compared with those without CVC, and CVC was correlated with heart failure, suggesting that CVC may be a marker of cardiovascular events. Similarly, some studies [1,2] have indicated that CVC itself is a superior predictor of clinical outcomes in ESKD patients and is closely associated with an increased risk of cardiovascular events and all-cause mortality. Taken together, these findings indicated that CVC might reflect a poor clinical prognosis in ESKD patients.

In summary, this cross-sectional study showed that CVC was a common complication and a danger signal for cardiovascular events in ESKD patients on combination therapy of hemodialysis and hemodiafiltration. The presence of diabetes, increased pulse pressure, long dialysis duration, hypoalbuminemia and high serum calcium levels were independent risk factors for CVC in ESKD patients on MHD.
